# 

**Published:** 1887-03

**Authors:** 


					﻿BOOKS AND PAMPHLETS RECEIVED.
Homoeopathic League Tracts:
No. 9. Allopathy Judged by its Professors.
No. 10. Eminent Medical Converts to Homoeopathy.
Report on Diseases of the Rectum. By Joseph M.
Mathews, M. D.
Sterility: Management of the Secundines. Wm.
H. Wathen, M. D.
The Pacific Record of Medicine and Pharmacy.
Published in both Spanish and English.
The Journal of Dietetics. Cleveland, Ohio. Pub-
lished quarterly.
President’s Address at Tenth Annual Meeting of
Detroit Medical and Library Association. By C. J.
Lunday, A. M., M. D.
NOTICE.
All articles for publication, books for review, business communications, etc.,
must be sent to Editors, 1123 Spruce Street, Philadelphia.
Subscribers will please notice that this Journal will be sent until arrears
are paid and it is ordered discontinued.
■ Subscribers, Contributors, and Exchanges,
please TAKE NOTICE. The Office of THIS
JOURNAL has been removed to 1123 Spruce
Street, where all communications must be sent.
All subscriptions are due in advance; subscribers will therefore
please forward their subscriptions at once; let each subscriber see
that his account is settled promptly.
Subscribers are respectfully requested to make all checks
and money-orders payable to THE HOM(EOPATHIC
PHYSIC PAN, and not to either of the Editors.
				

